# Infectious Diseases Telehealth Outcomes and Opportunities for Outpatient Parenteral Antimicrobial Therapy (OPAT) Patients Discharged From 18 Small Community Hospitals

**DOI:** 10.1093/ofid/ofaf703

**Published:** 2025-12-15

**Authors:** John J Veillette, Stephanie S May, Nick Tinker, Cecily Lucero, Erin Stahl, Jennifer Townsend, S Kyle Throneberry, Todd J Vento, Stephanie S Gelman, Allison M Butler, Brandon J Webb

**Affiliations:** Infectious Diseases Telehealth Service, Intermountain Health, Murray, Utah, USA; Department of Pharmacy, Intermountain Medical Center, Murray, Utah, USA; Infectious Diseases Telehealth Service, Intermountain Health, Murray, Utah, USA; Department of Pharmacy, Intermountain Medical Center, Murray, Utah, USA; Infectious Diseases Telehealth Service, Intermountain Health, Murray, Utah, USA; Department of Pharmacy, Intermountain Medical Center, Murray, Utah, USA; Division of Clinical Epidemiology and Infectious Diseases, Intermountain Medical Center, Murray, Utah, USA; Department of Pharmacy, Intermountain Homecare and Hospice, South Jordan, Utah, USA; Infectious Diseases Telehealth Service, Intermountain Health, Murray, Utah, USA; Division of Clinical Epidemiology and Infectious Diseases, Intermountain Medical Center, Murray, Utah, USA; Infectious Diseases Telehealth Service, Intermountain Health, Murray, Utah, USA; Division of Clinical Epidemiology and Infectious Diseases, Intermountain Medical Center, Murray, Utah, USA; Infectious Diseases Telehealth Service, Intermountain Health, Murray, Utah, USA; Division of Clinical Epidemiology and Infectious Diseases, Intermountain Medical Center, Murray, Utah, USA; Infectious Diseases Telehealth Service, Intermountain Health, Murray, Utah, USA; Division of Clinical Epidemiology and Infectious Diseases, Intermountain Medical Center, Murray, Utah, USA; Statistical Data Center, Intermountain Health, Murray, Utah, USA; Division of Clinical Epidemiology and Infectious Diseases, Intermountain Medical Center, Murray, Utah, USA

**Keywords:** community hospitals, infectious diseases, OPAT, tele-OPAT, telehealth

## Abstract

Infectious diseases telehealth (IDt) management of outpatient parenteral antimicrobial therapy (OPAT) across 18 small community hospitals was associated with similar rates of OPAT failure, lower rates of clinical failure and mortality, and higher likelihood of inpatient source control procedure compared to OPAT managed by other services. IDt program expansion might further improve care.

Outpatient parenteral antimicrobial therapy (OPAT) outcomes are optimized when overseen by infectious diseases (ID) physicians and pharmacists [1]; however, many small community hospitals (SCHs, <200 beds) lack access to such expertise [2]. Providing OPAT services via telehealth (Tele-OPAT) can improve access to ID clinicians, but limited outcomes data are available to guide structure, workflow, and staffing of Tele-OPAT programs [3–5]. Herein, we describe implementation, outcomes, and improvement opportunities for a Tele-OPAT program serving 18 SCHs within the Intermountain Health (IH) system, a nonprofit integrated network of 23 hospitals, 38 urgent cares, and 200 primary care clinics in Utah and Idaho.

## METHODS

The ID telehealth (IDt) service was implemented in 2017 to provide remote inpatient ID consultation and antimicrobial stewardship support to 16 SCHs [6]. By 2022, IDt coverage had grown to 18 SCHs (total 758 beds), inpatient staffing had increased to 2.6 FTE IDt physicians, and 2.0 FTE IDt pharmacists, and an acute outpatient need had arisen in the form of numerous OPAT discharges (estimated 500 total per year for all 18 SCHs). Inpatient IDt consultation relied on primary team request and was not mandatory for an OPAT discharge. Furthermore, there was a lack of resources for predischarge review or outpatient management of all potential OPAT patients. This led to variability in OPAT practices; therefore, we conducted this quality improvement project (exempted by the IH IRB) to evaluate OPAT outcomes and find opportunities for improvement.

We identified adults discharged from 18 SCHs on intravenous (IV) antibiotics between 5/2022 and 5/2023 and divided patients into 2 groups based on who managed the OPAT: IDt versus other services (non-IDt). All IDt OPAT patients had an inpatient IDt consultation via video visit or asynchronous electronic consultation and a follow-up outpatient IDt physician video visit within 2–4 weeks of discharge (either direct-to-patient or facilitated by nursing facility caregivers). IDt OPAT coordination and lab monitoring were performed by a nurse and 3 medical assistants, who were shared resources with the ID division at Intermountain Medical Center, while IDt pharmacists were available for drug-related questions as needed. Non-IDt patients had OPAT managed by a variety of other services (eg, orthopedics, general surgery, urology, and primary care), and only some had inpatient IDt consultation prior to non-IDt OPAT. Based on this workflow, we preplanned 3 subgroup analyses: one comparing non-IDt OPAT patients with and without inpatient IDt consultation, one stratifying by OPAT managing service, and one stratifying by OPAT duration (≤14 days vs longer).

Patient demographics and laboratory values were collected electronically from the enterprise data warehouse [7]; whereas, OPAT indication, microbiology, clinical course, IDt consultation details, outpatient follow-up visits, and outcomes within 90 days of discharge were collected via manual review of the electronic medical record (EMR). We excluded patients with OPAT duration < 3 days, OPAT prescribed with palliative intent, those who were lost to follow-up (no postdischarge EMR documentation during the 90-day follow-up period), or those who had OPAT managed outside the IH system. Data collection and retrospective clinical review of all OPAT patients was performed by the same ID pharmacist (J.J.V.).

The primary outcome was OPAT failure within 90 days, defined as a composite of clinical failure (recurrence/progression of infection at the primary site) or ED/hospital readmission due to OPAT complications (eg, line-related thrombosis and antibiotic-related neutropenia) [8, 9]. Secondary outcomes included all-cause 90-day readmissions, all-cause 90-day mortality, OPAT complications (including nonreadmissions), individual components of the primary outcome, and OPAT optimization opportunities [IV-to-PO conversion [10], antibiotic selection/dosing/duration (per IH guidelines), and source control during index admission (if applicable)].

Unadjusted pairwise comparisons were performed using Fisher's exact test for categorical data and the Mann–Whitney *U* test for continuous data. To mitigate indication bias, we calculated a propensity score for each patient's likelihood to receive IDt-managed OPAT (adjusted for infection type and discharging facility, Supplementary Table 1). Then, we used stabilized inverse probability treatment weighting (IPTW) in a logistic regression model accounting for Charlson comorbidity score and infection type to estimate the impact of IDt-management on OPAT failure, readmission, and mortality (R version 4.4.3) [11–13].

## RESULTS

Of 532 patients discharged on OPAT during the study period, 127 (24%) were excluded: 49 (9%) OPAT duration < 3 days, 43 (8%) lost to follow-up, 33 (6%) managed outside IH, and 2 (0.4%) prescribed OPAT with palliative intent. Of 405 included patients, 168 (41%) had IDt-managed OPAT (plus inpatient IDt consultation), and 237 (59%) had OPAT managed by other services [59% of whom (140/237) had inpatient IDt consultation]. There were numerous differences between groups, most notably that IDt OPAT patients had more bone/joint and endovascular infections, longer treatment durations, fewer urinary tract infections (UTIs), and fewer comorbidities (Table 1). IDt OPAT patients were more likely to receive daptomycin (than vancomycin) for dedicated Gram-positive coverage [47/60 (78%) versus 32/55 (58%), *P* = .03)], and more likely to have cultures obtained from the primary infection site [165/168 (98%) versus 214/237 (90%), *P* < .01] (Table 1). Among patients whose infection had a controllable source [ie, could be drained, debrided, or removed], IDt OPAT patients were more likely to have an inpatient source control procedure [105/131 (80%) versus 76/125 (61%), *P* < .01].

**Table 1.** Demographics and OPAT Management

	All Patients (*N* = 405)	IDt OPAT (*n* = 168)	Non-IDt OPAT (*n* = 237)	*P* Value
Age, median (IQR)	66 (53–75)	65 (53–72)	67 (55–76)	.11
Charlson comorbidity score, median (IQR)	6 (4–10)	5 (3–8)	8 (4–11)	**<.01**
Male gender^a^	201 (50)	94 (56)	107 (45)	**.03**
OPAT Indication (infection type)				
Bone or joint	163 (40)	108 (64)	55 (23)	**<.01**
Urinary tract infection (UTI)	87 (21)	9 (5)	78 (33)	**<.01**
Skin and soft tissue infection (SSTI)	43 (11)	11 (7)	32 (14)	**.03**
Intra-abdominal	24 (6)	3 (2)	21 (9)	**<.01**
Respiratory	21 (5)	5 (3)	16 (7)	.11
Endovascular	20 (5)	18 (11)	2 (1)	**<.01**
Bacteremia unknown source	20 (5)	7 (4)	13 (5)	.65
Line-related bacteremia	20 (5)	3 (2)	17 (7)	**.02**
Other	7 (2)	4 (2)	3 (1)	.46
Primary Organism				
*Staphylococcus aureus*	119 (29)	74 (44)	45 (19)	**<.01**
*Streptococcus* or *Enterococcus* spp	71 (18)	32 (19)	39 (16)	.51
Other Gram-positive organisms	31 (8)	16 (10)	15 (6)	.26
Enterobacterales	70 (17)	10 (6)	60 (25)	**<.01**
Other Gram-negative organisms	56 (14)	16 (10)	40 (17)	**.04**
Culture-negative	32 (8)	17 (10)	15 (6)	.19
No cultures obtained from infection site	26 (6)	3 (2)	23 (10)	**<.01**
OPAT Location				
Home health	221 (55)	113 (67)	108 (46)	**<.01**
Skilled nursing facility (SNF) or LTACH	106 (26)	33 (20)	73 (31)	**.01**
Infusion center	78 (19)	22 (13)	56 (24)	**.01**
OPAT duration				
≤14 d	219 (54)	30 (18)	189 (80)	**<.01**
>14 d	186 (46)	138 (82)	48 (20)	**<.01**
Median days (IQR)	14 (10–42)	39 (28–42)	11 (7–14)	**<.01**
OPAT regimen^b^				
β-lactam	330 (81)	134 (80)	196 (83)	.52
Daptomycin	79 (20)	47 (28)	32 (14)	**<.01**
Vancomycin	36 (9)	13 (8)	23 (10)	.60
Source control prior to discharge^c^	181/256 (71)	105/131 (80)	76/125 (61)	**<.01**

Bolded values are statistically significant (*P*<0.05); Abbreviations: OPAT, outpatient parenteral antimicrobial therapy; IDt OPAT, OPAT managed by the infectious diseases telehealth service; non-IDt OPAT, OPAT managed by any other service besides IDt; LTACH, long-term acute care hospital.

^a^Data are listed as number (percent) unless otherwise specified.

^b^Daptomycin and vancomycin are mutually exclusive, but β-lactam is not (eg, patient could have received a β-lactam + daptomycin). The most commonly prescribed β-lactams across both groups were ceftriaxone (34%), cefazolin (18%), and ertapenem (15%).

^c^Denominator was limited to patients whose source of infection was controllable (ie, could be drained, debrided, or removed).

The 90-day OPAT failure rate was lower in the IDt group (33% vs 36%) but was not statistically significant (Table 2). IDt OPAT failures were primarily driven by readmissions from OPAT complications, whereas non-IDt OPAT failures were driven by a significantly higher incidence of recurrent/worsening infections (Table 2). All-cause readmissions and mortality were lower in the IDt group with only the latter difference being statistically significant (Table 2). These results did not significantly change when stratifying by service managing the OPAT (Supplementary Table 2), OPAT duration (Supplementary Table 3), or inpatient IDt consultation prior to non-IDt OPAT (Supplementary Table 4), although these comparisons were limited by small sample size. The stabilized propensity-weighted logistic regression model was limited by small sample size and low event rate but yielded similar results to the unadjusted analysis: IDt OPAT demonstrated reduced failure rates but was not significantly different [odds ratio, OR 0.97 (95% CI .88–1.07)], and all-cause mortality was significantly lower in the IDt group [OR 0.94 (.90–.99)].

**Table 2.** 90-Day OPAT Outcomes

	All Patients (*N* = 405)	IDt OPAT (*n* = 168)	Non-IDt OPAT (*n* = 237)	Unadjusted *P* Value	Inverse Probability of Treatment Weighting (IPTW)^a^
Primary outcome					
OPAT failure^b,c^	140 (35)	55 (33)	85 (36)	.53	OR 0.97 (.88–1.07)
Clinical failure^d^	89 (22)	27 (16)	62 (26)	.**02**	OR 0.94 (.86–1.02)
Readmission for OPAT complication	66 (16)^e^	33 (20)	33 (14)	.13	OR 0.97 (.88–1.07)
Secondary outcomes					
All-cause unplanned readmission	200 (49)	76 (45)	124 (52)	.19	OR 1.02 (.92–1.14)
All-cause mortality	22 (5)	4 (2)	18 (8)	.**03**	**OR 0.94 (.90–.99)**
Any OPAT complication^f^	123 (30)	59 (35)	64 (27)	.10	
Adverse drug reaction (ADR)	94 (23)	45 (27)	49 (21)	.15	
Line-related complication	40 (10)	23 (14)	17 (7)	.**04**	

Abbreviations: OPAT, outpatient parenteral antimicrobial therapy; IDt OPAT, OPAT managed by the infectious diseases telehealth service; non-IDt OPAT, OPAT managed by any other service besides IDt.

^a^Listed as odds ratio (95% CI).

^b^Data are listed as number (percent).

^c^Reasons for OPAT failure are not mutually exclusive.

^d^Recurrent or worsening infection at primary site.

^e^Adverse drug reactions (ADRs) contributed to 48/66 (73%) of the overall readmissions for OPAT complication. The most common ADRs leading to readmission were acute kidney injury/electrolyte abnormalities (27%), cytopenia (10%), and allergic reaction (8%).

^f^Regardless of whether it led to readmission; causes of OPAT complications are not mutually exclusive.

Among all 405 patients, OPAT optimization opportunities fell predominantly in the non-IDt OPAT group (Supplementary Table 5). This was consistent among the 140 OPAT failures: 95/140 (68%) had optimization opportunities and 62 (65%) of these 95 fell in the non-IDt OPAT group. Details regarding potential contributing factors to these 95 OPAT failures are as follows: 55 (58%) inadequate source control, 24 (25%) suboptimal antibiotic selection/dose/duration, and 16 (17%) missed IV-to-PO conversions (which might have avoided a line-related complication). Supplementary Table 6 provides an additional breakdown of OPAT outcomes by care site.

## DISCUSSION

There was no difference in the composite OPAT failure rate between groups, a finding which was driven by OPAT complications from longer antibiotic durations (eg, for endovascular, bone/joint infections) in the IDt group versus higher clinical failures in the non-IDt group. IDt OPAT was associated with lower mortality and decreased readmissions compared to non-IDt OPAT; however, the latter difference was not statistically significant. It is possible these outcomes were driven by higher likelihood of OPAT optimization (source control, antimicrobials, IV-to-PO conversions) and infection site culture obtainment in the IDt group, which have been associated with improved OPAT outcomes [8, 9, 14]. Our 90-day failure rate for IDt OPAT (33%) was similar to 30-day failure rates reported in other OPAT studies (21–38%) [14, 15] suggesting that Tele-OPAT might be a safe and effective option for hospitals lacking on-site access to ID clinicians. Importantly, we identified several key interventions to address improvement opportunities and hopefully mitigate future OPAT failures: request an IDt OPAT pharmacist FTE (antimicrobial optimization and IV-to-PO conversions), request IDt physician FTEs (increase IDt ownership of OPAT patients), push for more early/direct communications with our surgical colleagues (source control), and engage with care managers to optimize the predischarge OPAT screening process (particularly for non-IDt OPAT patients).

Our study has several limitations. Data collection was limited to EMR documentation: we were unable to capture adverse events or therapy changes that were not documented, nor were we able to capture time to intervention on abnormal labs or adverse events by the IDt versus non-IDt teams. We did not capture IV access type during data collection. While we screened for OPAT failures within 90 days, it is possible that some patients had outcome-related events after the evaluation period. Reviewers were not blinded to the study objectives, and healthcare encounters outside of our system were not captured. Notably, all 18 SCHs were on the same EMR, which limits application of our results to hospitals providing telehealth services across different EMRs. While not explored in this study, EMR optimization is essential to improve transitions of care and OPAT workflow. Small sample size limited our ability to find differences between groups and to separate out the impact of inpatient IDt consult versus Tele-OPAT. It is possible that unmeasured confounders influenced the findings, although we made extensive efforts to adjust for imbalances between groups including propensity weighting, multivariate logistic regression, and exploratory subgroup analyses.

In summary, IDt management of OPAT was associated with improved outcomes for patients discharged from 18 SCHs. Based on these data, we have requested additional IDt pharmacist and physician resources and plan to make several OPAT-related practice changes, which we believe will further improve care.

## Supplementary Material

ofaf703_Supplementary_Data

## Supplementary Data

Supplementary materials are available at *Open Forum Infectious Diseases* online. Consisting of data provided by the authors to benefit the reader, the posted materials are not copyedited and are the sole responsibility of the authors, so questions or comments should be addressed to the corresponding author.
