# Supplementary material for: Infectious Diseases Telehealth Outcomes and Opportunities for Outpatient Parenteral Antimicrobial Therapy (OPAT) Patients Discharged From 18 Small Community Hospitals
Source: Open Forum Infect Dis. 2025 Dec 15;12(12):ofaf703. doi: 10.1093/ofid/ofaf703 (PMC12702615; doi:10.1093/ofid/ofaf703)
Supplement: ofaf703_Supplementary_Data [file ofaf703_supplementary_data.docx]

**SUPPLEMENTAL MATERIAL**

**Supplemental Table S1. Propensity model predicting likelihood of IDt-managed OPAT**

| **Variable** | **Odds Ratio (95% CI)** | **P-value** |
| --- | --- | --- |
| (Intercept) | 0.40 (0.15-1.01) | 0.05 |
| Bacteremia/endovascular infection | 2.25 (1.27-4.06) | 0.01 |
| Bone/joint infection | 1.49 (0.62-3.54) | 0.36 |
| Intra-abdominal infection | 0.08 (0.02-0.33) | <0.01 |
| All other infection types | 0.09 (0.04-0.21) | <0.01 |
| Discharging facility  Smallest rural sites (<25 beds) vs Hospitals 1/2^1^  Medium sites (25-150 beds) vs Hospitals 1/2 | 0.58 (0.19-1.62)  5.26 (2.91-9.87) | 0.31  <0.01 |

^1^Hospitals 1 and 2 were identified as least likely to request an ID consult and were used as a basis for comparison

**Supplemental Table S2. 90-day OPAT outcomes stratified by managing service**

| **OPAT service** | **OPAT patients** | **OPAT failures^2^** |
| --- | --- | --- |
| ID telehealth service | 168 | 55 (33) |
| Primary care physician | 89 | 29 (33) |
| SNF or LTACH physician | 55 | 16 (29) |
| Podiatry | 23 | 9 (39) |
| Orthopedics | 16 | 6 (38) |
| General Surgery | 15 | 8 (53) |
| None^1^ | 15 | 6 (40) |
| Urology | 10 | 5 (50) |
| Nephrology | 7 | 2 (29) |
| Wound care | 3 | 1 (33) |
| Bone marrow transplant | 1 | 1 (100) |
| Ear, nose, and throat (ENT) | 1 | 0 (0) |
| Gastrointestinal (GI) | 1 | 1 (100) |
| Thoracic surgery | 1 | 1 (100) |

^1^For these 15 patients, it was not evident on manual chart review that anyone was managing OPAT

^2^Data are listed as number (percent)

**Supplemental Table S3. 90-day OPAT outcomes stratified by treatment duration**

| **OPAT Duration ≤ 2 weeks** | **All Patients^1^**  **(n = 219)** | **IDt OPAT**  **(n = 30)** | **Non-IDt OPAT**  **(n = 189)** | **P-value** |
| --- | --- | --- | --- | --- |
| OPAT Failure | 75 (34) | 8 (27) | 67 (35) | 0.41 |
| All-cause unplanned readmission | 117 (53) | 12 (40) | 105 (56) | 0.12 |
| All-cause mortality | 14 (6) | 0 (0) | 14 (7) | 0.23 |
| Adverse drug reaction | 39 (18) | 4 (13) | 35 (19) | 0.61 |
| Line-related complication | 14 (6) | 1 (3) | 13 (7) | 0.70 |
|  |  |  |  |  |
| **OPAT Duration > 2 weeks** | **All Patients**  **(n = 186)** | **IDt OPAT**  **(n = 138)** | **Non-IDt OPAT**  **(n = 48)** | **P-value** |
| OPAT Failure | 65 (35) | 47 (34) | 18 (42) | 0.73 |
| All-cause unplanned readmission | 83 (45) | 64 (46) | 19 (44) | 0.50 |
| All-cause mortality | 8 (4) | 4 (3) | 4 (9) | 0.21 |
| Adverse drug reaction | 55 (30) | 41 (30) | 14 (33) | 1.00 |
| Line-related complication | 26 (14) | 22 (16) | 4 (9) | 0.23 |

^1^Data are listed as number (percent)

**Supplemental Table S4. 90-day OPAT outcomes stratified by inpatient IDt consultation**

|  | **Inpatient IDt consult**  **+ IDt OPAT^1^**  **(n = 168)** | **Inpatient IDt consult**  **+ Non-IDt OPAT**  **(n = 140)** | **No IDt consult**  **+ Non-IDt OPAT**  **(n = 97)** |
| --- | --- | --- | --- |
| OPAT Failure | 55 (33) | 50 (36) | 35 (36) |
| All-cause unplanned readmission | 76 (45) | 70 (50) | 54 (56) |
| All-cause mortality | 4 (2) | 10 (7) | 8 (8) |

^1^Data are listed as number (percent)

**Supplemental Table S5. OPAT optimization opportunities (regardless of outcome, success vs failure)**

|  | **Inpatient IDt consult**  **+ IDt OPAT^1^**  **(n = 168)** | **Inpatient IDt consult**  **+ Non-IDt OPAT**  **(n = 140)** | **No IDt consult**  **+ Non-IDt OPAT**  **(n = 97)** |
| --- | --- | --- | --- |
| IV-to-PO conversion opportunity during OPAT course | 48 (29) | 33 (24) | 43 (44) |
| Suboptimal antibiotic selection, dosing, or duration | 17 (10) | 18 (13) | 25 (26) |
| Lack of source control during index admission^2^ | 26/131 (20) | 26/77 (34) | 25/48 (48) |

^1^Data are listed as number (percent)

^2^Denominators for this row are limited to patients who had a controllable source of infection (i.e. could be removed, drained, or debrided)

**Supplemental Table S6. 90-day OPAT outcomes stratified by care site**

|  | **Home Health^1^**  **(n = 221)** | **Infusion Center**  **(n = 78)** | **Skilled Nursing Facility**  **(n = 98)** | **Long-term**  **Acute Care Hospital**  **(n = 8)** |
| --- | --- | --- | --- | --- |
| OPAT Failure | 81 (37) | 25 (32) | 30 (31) | 4 (50) |
| All-cause unplanned readmission | 114 (52) | 30 (38) | 51 (52) | 5 (63) |
| All-cause mortality | 5 (2) | 5 (6) | 11 (11) | 1 (13) |

^1^Data are listed as number (percent)
